# Beyond RNA-binding domains: determinants of protein–RNA binding

**DOI:** 10.1261/rna.080026.124

**Published:** 2024-12

**Authors:** Inbal Zigdon, Miri Carmi, Sagie Brodsky, Zohar Rosenwaser, Naama Barkai, Felix Jonas

**Affiliations:** 1 Department of Molecular Genetics, Weizmann Institute of Science, Rehovot 76100, Israel; 2 School of Science, Constructor University, 28759 Bremen, Germany

**Keywords:** intrinsically disordered regions (IDRs), RNA binding domains (RBDs), RNA binding proteins (RBPs), *S. cerevisiae*, mRNA binding proteins (mRBPs)

## Abstract

RNA-binding proteins (RBPs) are composed of RNA-binding domains (RBDs) often linked via intrinsically disordered regions (IDRs). Structural and biochemical analyses have shown that disordered linkers contribute to RNA binding by orienting the adjacent RBDs and also characterized certain disordered repeats that directly contact the RNA. However, the relative contribution of IDRs and predicted RBDs to the in vivo binding pattern is poorly explored. Here, we upscaled the RNA-tagging method to map the transcriptome-wide binding of 16 RBPs in budding yeast. We then performed extensive sequence mutations to distinguish binding determinants within predicted RBDs and the surrounding IDRs in eight of these. The majority of the predicted RBDs tested were not individually essential for mRNA binding. However, multiple IDRs that lacked predicted RNA-binding potential appeared essential for binding affinity or specificity. Our results provide new insights into the function of poorly studied RBPs and emphasize the complex and distributed encoding of RBP–RNA interaction in vivo.

## INTRODUCTION

Gene expression depends on transcription factors (TFs) that bind regulatory regions on DNA and RNA-binding proteins (RBPs) that regulate the transcribed RNA. RBPs guide the various maturation steps of transcribed RNAs, including pre-mRNA splicing, cleavage, and polyadenylation, and further act on the mature transcripts to regulate their stability, localization, editing, or translation. The human genome contains over a thousand RBPs (Gerstberger et al. 2014) and many of these are associated with diseases such as neurodegeneration, autoimmunity, and cancer, underscoring their critical role in cell physiology (Lukong et al. 2008; Cooper et al. 2009; Castello et al. 2013; Gebauer et al. 2020).

Structural analysis has served as the main tool for describing mechanisms of RBP interaction with RNAs (Han and Nepal 2007; Pérez-Cano and Fernández-Recio 2010). Canonical RNA-binding domains (RBDs) are classified into conserved families adopting characteristic 3D structures that interact with RNA by contacting specific RNA bases or recognizing particular RNA structures (Draper 1999; Jones et al. 2001; Mackereth and Sattler 2012). Among these RBD families, the RNA recognition motif (RRM) is the most common fold, appearing in an estimated ∼1% of human proteins. Other domain families include the K-homology (KH), DEAD/DEAH helicase, and zinc finger domains, as well as ∼40 additional domain families of lesser abundance (Gerstberger et al. 2014).

A typical RBD binds RNA at low affinity and specificity, recognizing only a few nucleotides (Lunde et al. 2007; Helder et al. 2016; Corley et al. 2020; Jolma et al. 2020; Ottoz and Berchowitz 2020). Individual RRM domains, for example, bind variable motifs of two to eight bases (Maris et al. 2005; Lunde et al. 2007; Cléry and Allain 2011; Corley et al. 2020), and each repeat of the Pumilio homology domain (PUM-HD), consisting of a helix of 36 amino acids (AAs), recognizes just one base (Wang et al. 2018; Zhao 2018). The need to increase binding affinity and specificity may explain the abundance of RBPs containing multiple RBDs of the same or of different families (Lunde et al. 2007; Cléry and Allain 2011; Mackereth and Sattler 2012). PUM-HDs are comprised of eight sequential repeats, for example, thereby binding to an eight-base motif. Other well-studied examples include the PTB protein, which combines four RRM domains (Oberstrass et al. 2005), and FUS, which contains a combination of an RRM, a zinc finger, and several RGG repeats (Loughlin et al. 2019).

RBDs appearing within the same protein are often linked through disordered linkers, and these can contribute to binding affinity and specificity in at least two ways. First, the linker controls the maximal distance and relative orientation of the various RBDs and the extent to which they depend on each other (Shamoo et al. 1995; Lunde et al. 2007; Mackereth and Sattler 2012; Helder et al. 2016). Second, in some cases, the disordered linkers form direct contact with the RNA (Maris et al. 2005; Cléry and Allain 2011), an interaction that can involve a disorder-to-order transition (Handa et al. 1999; Pérez-Cañadillas 2006).

Besides the RBDs and their connecting linkers, RBPs are enriched in extended intrinsically disordered regions (IDRs) (Castello et al. 2012; Neelamraju et al. 2015). This is perhaps most notable in RBPs that lack predicted RBDs of known families, as revealed by recent unbiased screens (Hentze et al. 2018; Baltz et al. 2012; Castello et al. 2016; Beckmann et al. 2015). Functionally, IDRs can contribute to the formation of large molecular assemblies containing proteins and RNAs (Uversky and Fink 2004; Toretsky and Wright 2014; Uversky et al. 2015; Wu and Fuxreiter 2016) such as stress granules (Kedersha et al. 2013) and processing bodies (P-bodies) (Eulalio et al. 2007; Jain and Parker 2013). Short, repeated motifs often embedded within IDRs can also contribute to nonspecific RNA binding, as shown for basic stretches (Arginine-rich motifs, ARMs) (Bayer et al. 2005) or SR/RG (Phan 2011; Castello 2012; Järvelin et al. 2016; Hentze et al. 2018). Yet, most extended IDRs within RBPs remain uncharacterized.

To better understand the relative contribution of this composite of folded RBDs and IDRs to the transcriptome-wide in vivo binding and specificity of RBPs, we provide here a detailed analysis of RBP binding determinants in *Saccharomyces cerevisiae*. For this, we upscale the RNA-tagging method (Lapointe et al. 2015) and apply it to define the mRNA-binding profile of 16 mRNA-binding proteins (mRBPs) (out of 34 tested). We then proceed with extensive truncations of eight of these mRBPs, distinguishing binding determinants within predicted RBDs and adjacent IDRs lacking predicted domains.

## RESULTS

### Predicted RBDs and IDRs occupy a large fraction of the mRBP sequences and are conserved throughout evolution

To determine the relative contribution of different predicted RBDs as well as the potential contribution of linkers and extended IDRs within RBPs to RNA binding in vivo, we first wished to obtain a proteome-wide view of the predicted RBD and IDR composition of RBPs and their distribution within each protein sequence and also to compare it to that of TFs, composed of DNA-binding domains (DBDs) and IDRs, which both have a role in binding promoter targets in vivo (Brodsky et al. 2020; Kumar et al. 2023).

**FIGURE 1.**
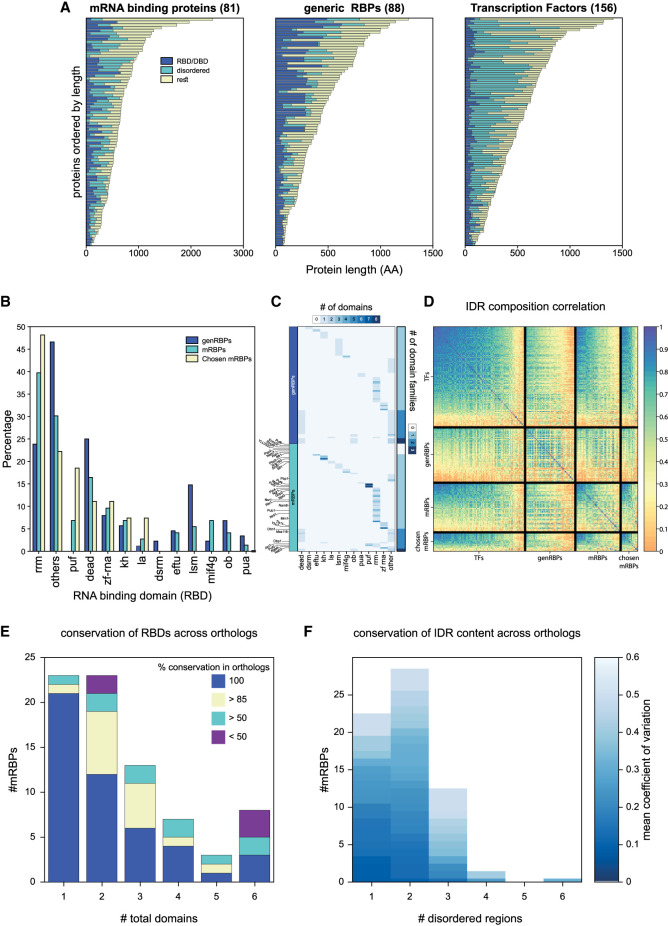
Predicted RBDs and IDRs occupy a large fraction of the mRBP sequences and are conserved throughout evolution. (*A*) Number of residues in RNA- or DNA-binding domains (RBD/DBD), disordered regions and the rest of the protein for mRBPs, genRBPs, and TFs. Proteins are ordered according to length in amino acids (AA). (*B*) Bar graph showing the percentage of genRBPs, mRBPs and chosen mRBPs containing the 12 indicated common RBDs or “others.” (*C*) Heatmap showing the type of RBDs (columns) in each of the genRBPs and mRBPs (rows); color indicates the number of domains. *Right* panel indicates the number of different domain families each RBP has. Names of the mRBPs chosen for experimental analysis are shown. (*D*) Pairwise correlation of AA composition between TFs, genRBPs, mRBPs, and chosen mRBPs. Of note, mRBPs also contain the chosen mRBPs. (*E*) Bar graph showing the number of mRBPs with 1–6 (or more) RBDs classified by the conservation of their RBD organization across all orthologs (i.e., type, number, and relative position). (*F*) Bar graph showing the number of the different mRBPs with 1–6 or more disordered regions. Color indicates the mean variation coefficient across the IDR regions of all orthologs.

The budding yeast genome contains 519 proteins annotated as RBPs (Cherry et al. 2012). The majority of these form large ribo-protein complexes such as the ribosome or spliceosome. We did not include these in our analysis but focused on the 169 RBPs associated with mRNAs (i.e., mRBPs), or other RNAs (Cherry et al. 2012; Potter et al. 2018). Budding yeast contains 88 RBPs which are not associated with large enzymatic complexes (hereafter: generic RBPs, genRBPs), and 81 mRBPs, adding up to 169 proteins, a number that is comparable to that of specific TFs: 156 (Fig. 1A; Cherry et al. 2012; Weirauch et al. 2014 ). The group of mRBPs is most interesting to us, as their role in gene regulation might entail binding to a subset of the ∼6000 mRNA transcripts and identifying specific binding determinants. Sequences of mRBPs are, on average, longer than genRBPs (604 vs. 392 residues median length), and are comparable to TFs (626 residues median) (Fig. 1A; Supplemental Fig. S1A; Cherry et al. 2012; Potter et al. 2018).

We defined RBDs within mRBPs, and DBDs within TFs, based on Pfam (Mistry et al. 2021) annotation, generated through multiple sequence alignments of known family members (see Materials and Methods). Overall, predicted RBDs within RBPs can largely be classified into 12 common families (Fig. 1B). The most common RBD families were the RRM in mRBPs (37%) (Fig. 1B) and DEAD box helicase in genRBPs (25%) (Fig. 1B; Potter et al. 2018).

When examined individually, RBDs were longer than DBDs (85 vs. 36 AAs, respectively). Furthermore, when considering the full protein, multiple domains (two or more) were significantly more frequent within RBPs than within TFs. Thus, while multiple DBDs appear in only 20 TFs, multiple RBDs, usually from the same family, are present in 59% of mRBPs and 39% of the genRBPs (Fig. 1C; Supplemental Fig. S1C), with a median number of 2 RBDs per mRBPs (Fig. 1C, see Materials and Methods).

The multiplicity of RBDs within RBPs resulted in a significantly higher fraction of the RBP sequence being associated with RNA binding: annotated RBDs occupied, on average, 27% of the mRBP's sequence and 38% of the genRBPs, as compared to 13% of TF sequence being annotated as DBD (Fig. 1A; Supplemental Fig. S1B; Potter et al. 2018). Furthermore, these RBDs were most often distributed throughout the mRBP sequence rather than localized at a particular region, such that the predicted region occupied by RBDs spanned a range of 35% of protein sequence, as compared to 13% in TFs, and similar to the span of 44% of the genRBPs (Supplemental Fig. S1D; Potter et al. 2018).

A large fraction of each mRBP is, therefore, associated with mRNA binding through the multiplicity of RBDs distributed along the sequence. Since extended IDRs were also implicated in RNA binding, we next examined the fraction of RBP sequences annotated as IDRs. On average, 35% of the mRBPs’ sequence could be defined as IDR (Fig. 1A; Supplemental Fig. S1E; see Materials and Methods), and this was comparable to the fraction of IDRs within TFs (37%) (Fig. 1A; Supplemental Fig. S1E), but significantly higher than the fraction of IDRs within the genRBPs and the total proteome (16% and 17%, respectively) (Fig. 1A; Supplemental Fig. S1E; Mészáros et al. 2018). When considered together, 62% of the typical mRBP sequence was annotated as either RBD or IDR (Mészáros et al. 2018; Potter et al. 2018) (see Materials and Methods). In terms of AA composition, the IDRs of mRBPs were highly similar to the ones within TFs but were rather distinct from the ones found within the genRBPs (Fig. 1D).

We reasoned that if the multiplicity of RBDs and IDRs is of relevance for the RBP's function, it would be conserved across evolution. To test this, we compared the RBDs domain composition, individual RBDs sequence, and composition of extended IDRs between *S. cerevisiae* and related yeast species (Fig. 1E,F; Supplemental Fig. S2) up to 100 MY apart. We found a high level of conservation in both RBD composition, including identity, sequence, and location, with more than 80% of orthologs of most mRBPs sharing the same domain composition (Fig. 1E; Supplemental Fig. S2). Moreover, the presence and size of the associated IDRs were also conserved between orthologs with IDR conservation of most orthologs varying by less than 30% (Fig. 1F; Supplemental Fig. S2). We conclude that mRBPs commonly contain multiple RBDs, interspaced or flanked by extended IDRs, which together encompass a major fraction of their protein sequence.

### Selecting RBPs for experimental analysis

Our analysis above suggested that a large fraction of the mRBP sequence could contribute to RNA binding in vivo. We next wished to distinguish experimentally the relative contribution and potential interactions of predicted RBDs and IDRs within the same mRBP. For this, we first needed to select a subset of RBPs to analyze. We began by screening a large number of mRBPs. In our selection, we included mRBPs that contain different numbers of RBDs (Fig. 1C; Supplemental Fig. S1C), of different types (Fig. 1B,C), spread differently across the protein sequence, and include different fractions of IDRs (Supplemental Fig. S1E; Potter et al. 2018; Mészáros et al. 2018). We also included mRBPs that were successfully profiled previously using the RNA-tagging method. Based on these, rather loose, criteria, we chose 34 of the total 81 mRBPs for our initial screen (Fig. 1C).

### Mapping mRBP binding using the RNA-tagging method

Approaches for mapping genome-wide RBP–RNA interactions can be classified into two groups. First, common approaches are based on RNA immunoprecipitation (RIP) or cross-linking and immunoprecipitation (CLIP). Second, newer approaches identify RBP targets by fusing RBPs to RNA modifying enzymes and detecting those modifications through sequencing (Hafner et al. 2021). Although most commonly used, the RIP and CLIP methods require protein purification and are subjected to noise coming from protein fixation and from the low amounts of purified, UV-cross-linked complex. We, therefore, decided to adopt the RNA-tagging method, an RNA modification approach, in which the RBP of interest is fused to the poly(U) polymerase of *Caenorhabditis elegans* (Pup-2) (Lapointe et al. 2015). When recruited to RNA, Pup-2 adds a stretch of polyuridines to the 3′-end of the RNA. This method, therefore, allows the detection of all mRNAs bound by a given RBP of interest through high-throughput mRNA sequencing (Lapointe et al. 2015). Our optimized protocol detects poly(U) stretches specifically on target mRNAs, and further quantifies their abundance compared to the total mRNA pool (Supplemental Fig. S3).

**FIGURE 2.**
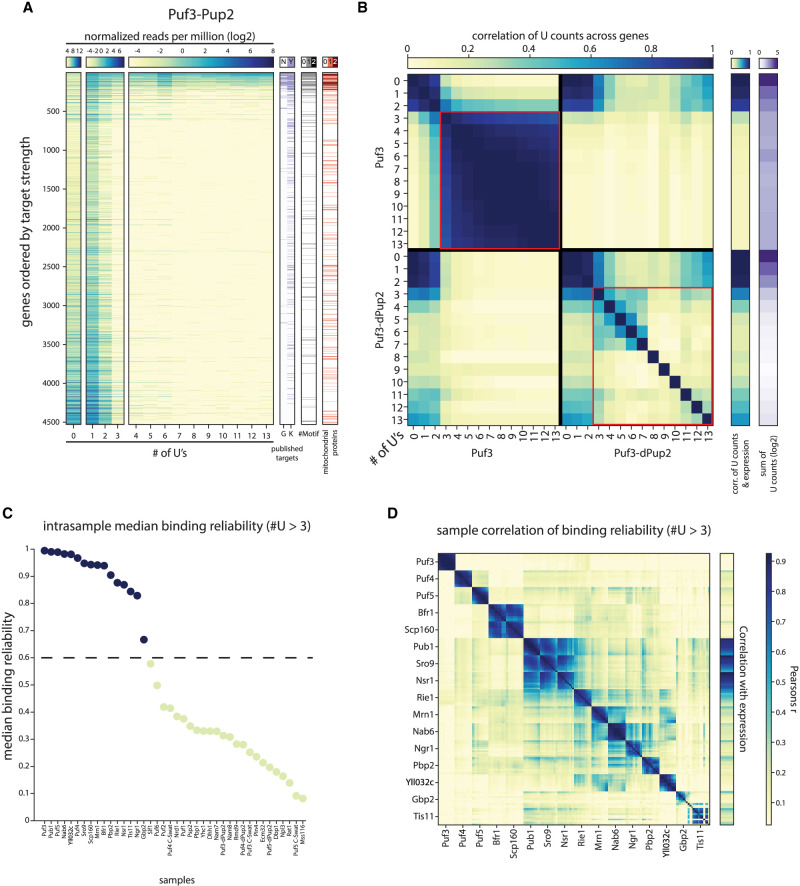
Mapping mRBP-binding profiles using the RNA-tagging method. (*A, left*) U-count matrix for Puf3-Pup2 strain, transcripts (rows) are ordered according to their modification level in log2 (binding score to expression ratio; see Materials and Methods). Color indicates the number of transcripts per million in log2 with the indicated number of Us (columns). (*Right*) Puf3 targets according to two separate publications (G, K) (Gerber et al. 2004; Kershaw et al. 2015), motif occurrences within the 3′ end, and mitochondrial GO. (*B, left* to *right*) Pairwise Pearson correlation of U counts across genes between U stretches of length 0–13 for Puf3-Pup2 and Puf3-dPup2 strains; correlation of U counts for indicated U stretch lengths with expression level in each strain (see Materials and Methods), sum of U counts across all genes for indicated U stretch lengths (0–13, log2). Marked in red: correlation of U counts for U stretches of length 4–13 across genes between themselves used to determine binding reliability (see Materials and Methods). (*C*) Median binding reliability (see Materials and Methods) for all mRBPs tested and dPup2 or nontagged (C-Swat) controls. Color indicates those mRBPs above the reliability threshold indicated by dotted line. (*D*) Pairwise Pearson correlation of U counts across genes between U stretches of length 4–13 (binding reliability), and correlation of U counts with expression for all strains with a median binding reliability above the threshold indicated in *C*.

Previous studies applied the RNA-tagging method for profiling the RBPs of the Pumilio RNA-binding protein family (Puf3-5) and the noncanonical RBP Bfr1 (Lapointe et al. 2015, 2017). We, therefore, tested our protocol first by profiling Puf3 (Fig. 2A). Fusing Puf3 to the Pup-2 enzyme led to the expected addition of poly(U) stretches of various lengths after the poly(A) tail of the known Puf3 targets (Gerber et al. 2004; Kershaw et al. 2015), most notably transcripts coding for mitochondrial proteins, and those that contained the known Puf3 binding motif (Fig. 2A).

Examining the length distribution of the added poly(U) stretches, we noted that short stretches of lengths 1–3 did not localize preferentially to the known Puf3 targets but were rather proportional to transcript abundance (Fig. 2A,B). Longer stretches did not correlate with expression but were tightly correlated among themselves (Fig. 2B), reflecting their strict association with Puf3 targets (Fig. 2A). Repeating the experiment in a control strain in which Puf3 was fused to catalytically inactive Pup-2 (dPup-2), we found that short, 1–3 poly(U) stretches are still observed and retain their correlation with expression, indicating that these U counts likely reflect sequencing errors (Fig. 2B). In contrast, longer stretches were now rare, and showed little, if any correlation among themselves indicating that they no longer appear on mRNAs of the same genes (Fig. 2B). Together, we conclude, within our RNA-tagging protocol, the correlated pattern of poly(U) stretches of lengths > 3 can be used as a measure for the reliability of the observed mRNA binding.

We next extended the analysis to 33 additional RBPs (see Fig. 1). Sixteen of the RBPs analyzed gave a reliable binding profile, as judged by the consistent patterns of long poly(U) stretches (Fig. 2C), and the reproducibility of these long poly(U) profiles between repeats (Supplemental Fig. S4). Comparing the poly(U) profiles among these sixteen RBPs revealed that most were largely distinct, indicating specific binding to different subsets of transcripts (Fig. 2D). The two interacting proteins Bfr1 and Scp160 gave practically the same profile, indicating their localization to the same set of transcripts (Fig. 2D). High correlations were also observed between Pub1, Sro9, and Nsr1, and, to a lesser extent, Mrn1, and Nab6 (Fig. 2D). Of note, none of the dPup-2 controls gave a consistent poly(U) pattern, as expected (Fig. 2C; Supplemental Fig. S4).

We note the large number of RBPs for which the RNA-tagging method did not generate a reliable profile. This reflects the limitations of this tagging method, which likely depends on stable binding to the 3′ UTR. Indeed, such failure characterized RBPs that act during rapid transcription-related processing, including splicing (Yhc1, Nam8, Psp2, and Mss116), nuclear export (Nab2), or 3′ end processing (Rat1, Nrd1, or Nam7) (Cherry et al. 2012).

### Binding profiles of RBPs provide new insights into their function

We next defined the set of target genes bound by each RBP in our data set. We considered that RBP specific target transcript molecules containing longer poly(U) stretches are, on average, bound for a longer fraction of time. To account for that, we measured binding using a weighted sum of poly(U) stretches longer than 3 Us, counting the overall number of uridines associated with each transcript (hereafter: binding score, see Materials and Methods).

**FIGURE 3.**
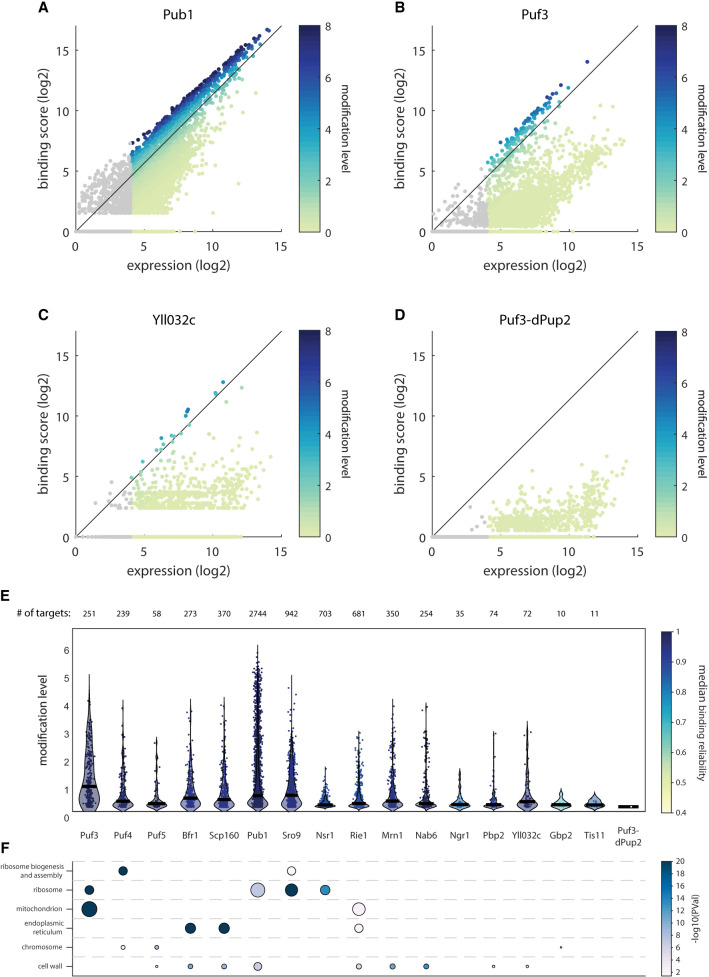
Binding profiles of RBPs provide new insights into their function. (*A*–*D*) Binding score as a function of expression (log2) for all genes in Pub1 (*A*), Puf3 (*B*), Yll032c (*C*), and Puf3-dPup2 (*D*). Color represents modification level (binding score to expression ratio; see Materials and Methods). (*E*) Distribution of modification level of targets of indicated mRBPs and Puf3-dPup2 control. Transcripts with a modification level higher than 0.15 were defined as targets. The number of targets for each RBP is indicated on *top*. Color represents the median binding reliability. (*F*) Significantly enriched (pVal < 0.05) GO groups among targets for mRBPs and Puf3-dPup2 control. Color and size indicate *P*-value and the number of targets in the GO-term, respectively.

Similar to Puf3, highly transcribed genes often included some poly(U) stretches, and this was seen, to some extent, in control samples carrying catalytically dead Pup-2 (Fig. 3A–D). This dependence on mRNA abundance, however, differed between mRBPs. In some cases, such as Pub1, mRNA abundance fully explained the observed binding score, suggesting limited specificity (Fig. 3A). In other cases, however, high binding scores were primarily observed at intermediately expressed genes, as exemplified by Puf3 and Yll032c (Fig. 3B,C).

Considering this quantitative dependence of binding score on transcript abundance, we decided to calculate the ratio of binding score to total abundance (hereafter: modification level). Based on the modification level, we defined, for each RBP, the set of target genes. The number of significantly modified targets ranged from ten (Gbp2) to 2744 (Pub1), while the median modification level ranged from 0.56 for Pub1 targets to 0.24 for Gbp2 (Fig. 3E). Of note, for Pub1 for example, this modification level corresponds to an average of 11% of target transcripts being poly(U) tailed with 4 uridine or more.

We next searched for enriched functions within these targets using gene ontology (GO) analysis (Cherry et al. 2012). This revealed the expected enrichment of mitochondrial genes within Puf3 targets (Lapointe et al. 2015), as well as the association of Puf4 with ribosome biogenesis genes (Fischer and Olivas 2018; Kochan et al. 2021) and of Puf5 with chromosomal function (Kochan et al. 2021). Bfr1 and Scp160 were both associated with ER functions, consistent with the subcellular localization of Bfr1 and Scp160 (Weidner et al. 2014) and existing mRNA tagging data (Lapointe et al. 2015). Rie1 was associated with both mitochondrial and ER functions, while Mrn1, Nab6, Pbp2, and Yll032c were associated with cell-wall (Fig. 3F; Cherry et al. 2012). Of note, the partial correlation between these cell-wall-associated RBPs (Figs. 2D and 3F) suggests their involvement in different aspects of cell-wall function. Pub1, Sro9, and Nsr1 were all enriched in transcripts coding for ribosomal proteins (Fig. 3F). Closer examination revealed that this enrichment resulted from the high expression levels of these transcripts, as also the modification level of these RBPs was largely explained by transcript abundance (Fig. 2D).

This expression-related binding score suggests limited specificity and a general role in translation. Consistently, Pub1 was shown to modulate stop-codon decoding during translation termination, a function that is required in all translated mRNAs, and the poorly characterized Sro9 localizes to polysomes (Sobel and Wolin 1999). Of note, Pub1 and Sro9 have a large number of targets (2744 and 942, respectively), which they bind strongly and with high (median) modification levels (0.56 and 0.58, Fig. 3E), while Nsr1 binds a large number of targets (703), but probably weaker as its modification level is half of that of Pub1 (0.23, Fig. 3E). A possible explanation for a weaker binding of Nsr1 to its targets could be its additional roles in rRNA processing and DNA binding (Cherry et al. 2012). We conclude that the RNA-tagging method provides reliable binding data describing the binding targets and strength for sixteen of the RBPs we have tested.

### Differential contribution of RBDs to binding affinity and specificity

**FIGURE 4.**
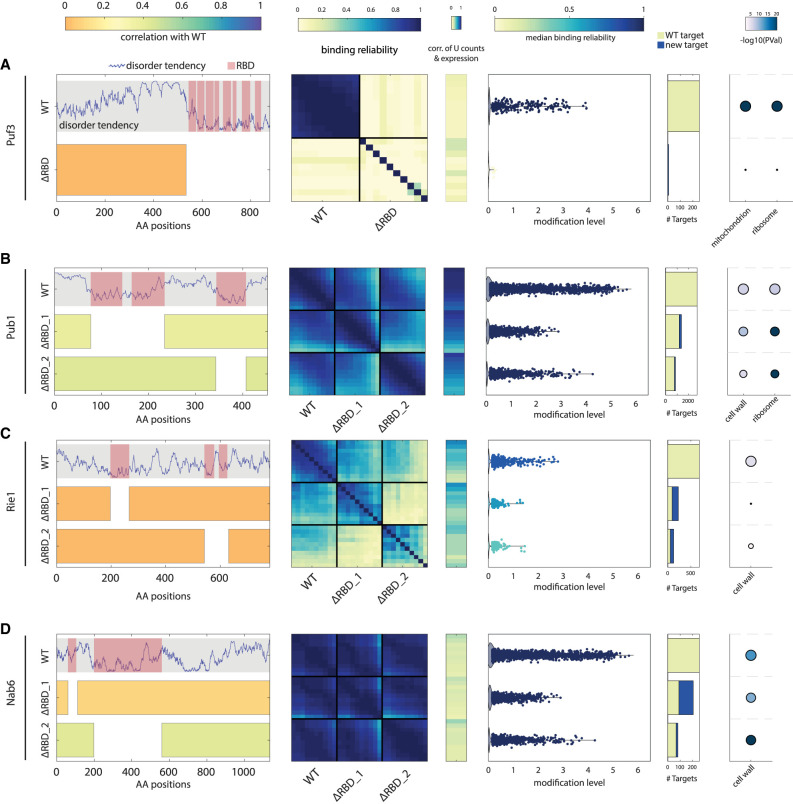
Differential contribution of RBDs to binding affinity and specificity. (*A, left* to *right*) Disorder tendency along the length of Puf3 with RBDs indicated in pink. Beneath, a representation of the residues kept in the RBD deletion strain; color indicates the correlation of the modification level in log2 between the mutant and WT strain. Heatmap of binding reliability for WT and RBD deletion strain (see Fig. 2B). Correlation of expression with U counts for U stretch length 4–13. Scatter showing the modification level of each target, color indicates median binding reliability. Bar graph of the number of targets classified into old or new with respect to WT targets. GO-term enrichment of WT and mutant targets (as in Fig. 3F). (*B*–*D*) As in (*A*) for Pub1, Rie1, and Nab6. See Supplemental Figure S5A–D for Scp160, Puf4, Puf5, and Mrn1.

Next, we examined the contribution of different regions within RBPs to their in vivo binding affinity and specificity. The RBPs that were successfully profiled included different combinations of RBDs. In some cases, all RBDs were localized to a single region of the protein, while in others the RBDs were dispersed across the sequence. We, therefore, chose eight RBPs that gave a reliable binding signal. In four of those, the RBDs were clustered together, flanked by long IDRs (Puf3, Puf4, Puf5, and Mrn1) (Fig. 4A; Supplemental Figs. S2A and S5B–D), and the other four included dispersed RBDs (Pub1, Rie1, Nab6, and Scp160) (Fig. 4B–D; Supplemental Figs. S2A and S5A).

As a first analysis, we considered the role of RBDs clustered in the same region, by deleting the full region. This deletion consistently led to a complete loss of binding signal, as indicated by the loss of correlations between poly(U) stretches of different lengths, and the lack of significantly bound genes (Fig. 4A; Supplemental Fig. S5B–D).

Next, we considered the four RBPs having dispersed RBDs and deleted each RBD-containing region individually. In all four cases, the binding signal for the original, WT targets was reduced, but not eliminated (Fig. 4B–D; Supplemental Fig. S5A). This reduction in binding signal has led to a loss of weakly bound targets. The two deletions within Pub1, for example, led to a loss of more than half of its targets with significant overlap between the deletions (Supplemental Fig. S9), and the remaining targets were also modified to a lower extent (modification level of 0.30 and 0.40, respectively, compared to 0.56 for WT) (Fig. 4B). In other cases, the effect was more severe: Rie1, for example, lost almost all of its targets after either one of its RBD deletions (Fig. 4C). However, it gained binding to targets not bound by the intact Rie1, and a similar gain of targets was seen also in Nab6 and Scp160 (Fig. 4C,D; Supplemental Fig. S5A). We conclude that in the case of dispersed RBDs, it is their combination that is required for binding and specificity, either by increasing binding strength (exemplified by Pub1) or by increasing specificity and preventing binding to other genes (as seen in Scp160 and Nab6). However, these combinatorial effects were not associated with the biological function of different targets for the RBD-deleted variants (Fig. 4; Supplemental Fig. S5, rightmost panel).

### mRBP-binding profiles depend on IDRs outside predicted RBDs

**FIGURE 5.**
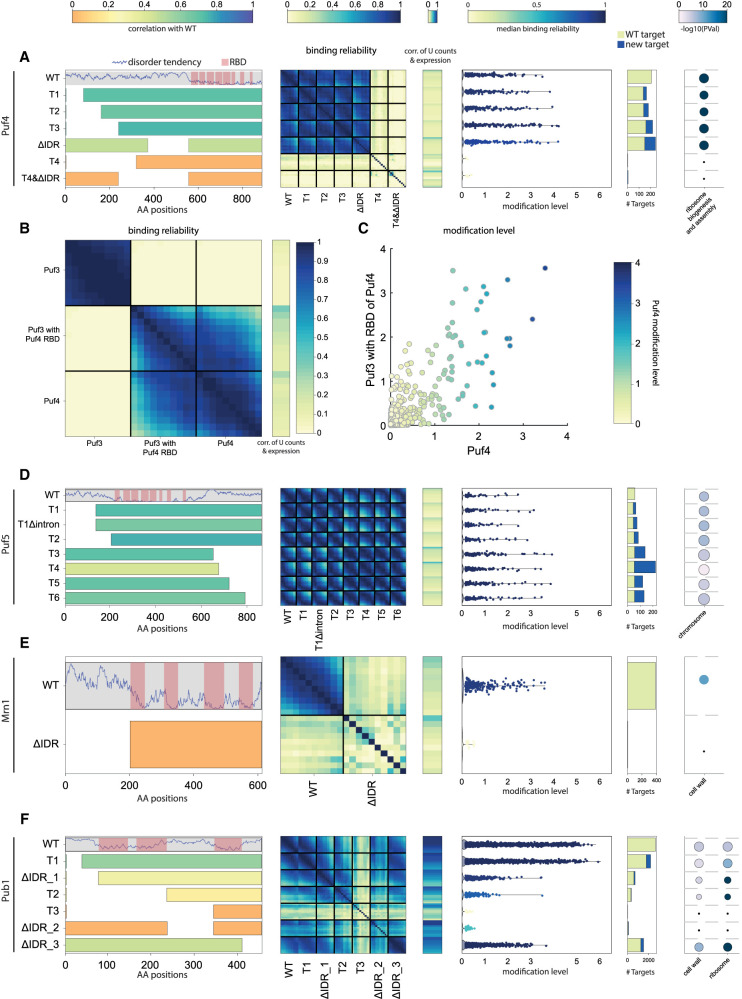
RBP's binding profiles may depend on IDRs outside predicted RBDs. (*A*) As in Figure 4A for the non-RBD deletion strain Puf4. (*B*) As in Figure 2D for Puf3, Puf4, and Puf3 with Puf4 RBD. (*C*) Scatter plot comparing modification level of all transcripts in Puf3 with Puf4 RBD and Puf4. Color indicates the modification level in Puf4. (*D*–*F*) As in (*A*) for Puf5, Mrn1, and Pub1. See Supplemental Figure S6A–D for Puf3, Nab6, Scp160, and Rie1.

In addition to containing multiple RBDs, the RBPs in our data sets also included extended IDRs. Motivated by the high fraction of IDRs within mRBPs, their conservation across species, and their AA composition similarity to that of IDRs within TFs, we asked whether these IDRs contribute to in vivo binding, by modulating binding strength or specificity. For this, we focused first on the three related mRBPs, Puf3-5. In all three proteins, the RBDs, which are essential for binding, occupy ∼200 to 400 of ∼800 residues sequence, while the rest of the sequence is of low complexity predicted to form extended IDRs (Fig. 5A,D; Supplemental Fig. S6A). We generated a series of deletions that gradually truncated the non-RBD regions of each protein, removing ∼40 to ∼100 residues at each step. All truncations were fused to the Pup-2 enzyme to allow profiling of the in vivo binding (Fig. 5A,D; Supplemental Fig. S6A).

The most significant effect was seen in Puf4, where a deletion of fewer than 400 residues from the beginning to the middle of the protein fully abolished the binding signal (Fig. 5A). Moreover, just deleting ∼300 highly disordered, partially overlapping, AAs in the middle of Puf4 had the same effect (Fig. 5A). In an attempt to differentiate between target specificity determinants and general binding determinants within the highly disordered non-RBD of Puf4, we tested whether the non-RBD of Puf3 with Puf4 RBD can also direct binding to Puf4 targets. As the non-RBD of Puf3 with Puf4 RBD localized to most of Puf4 targets in a similar pattern (although not identical) to that of Puf4 and not Puf3 (Fig. 5B,C), our results point to disordered general binding determinants in the non-RBD of Puf4.

Notably, in all other Puf3-5 non-RBD truncations, the IDR truncation did not abolish target binding (Fig. 5A,D; Supplemental Fig. S6A). However, specificity was reduced, as additional transcripts not modified by the intact RBP were now modified (Fig. 5A,D; Supplemental Fig. S6A). Generally, this binding to nontargets increased gradually as larger regions were truncated, indicating that specificity depends on multiple determinants distributed within the truncated IDRs. Therefore, the extended IDRs of Puf3-5 are all required for limiting binding to their respective targets, and this is done through multiple determinants distributed throughout the sequence.

To examine whether this contribution of IDRs extends to other RBPs in our data set, we next deleted non-RBD regions that flank, or separate the different RBD domains, considering both the IDRs and the structured regions. In all cases, these deletions reduced, or even abolished target binding. In the case of Mrn1, the removal of the extended IDR region flanking the RBDs-containing region led to a complete loss of binding (Fig. 5E), as did the removal of the extended IDR separating the two RBD-containing segments of Pub1 (Fig. 5F). In these cases, no new targets appeared, indicating that binding capacity was fully lost (Fig. 5E,F). In other cases, deletions of non-RBD regions, with varying tendencies of disorder, resembled more the deletion of individual RBDs, leading to a partial (Nab6 and Scp160) or full (Rie1) loss of targets, but the gain of some new targets in Nab6 and Scp160 (Supplemental Fig. S6B–D).

Of note, the changes in binding specificity can be indirect via changes in mRBP expression level. However, significant changes in mRBP's mRNA levels were rare and did not correlate with binding change (Supplemental Fig. S7). Similarly, Puf3 and Puf4 protein levels were only affected in a few variants and significant changes in binding were also observed without change in protein abundance (Supplemental Fig. S8).

We conclude that non-RBD regions within RBPs, most of which form extended IDRs, contribute to binding strength and specificity independent of their exact position in relation to the RBDs.

## DISCUSSION

In our study, we present the RNA-binding targets of 16 *S. cerevisiae* mRBPs using an upscaled version of the RNA-tagging method. Many targets of these profiled mRBPs have not been previously characterized. The binding profiles of these proteins highlight their central role in cell physiology which can be general or specific from protein secretion to mitochondria.

As not all of the attempted 34 proteins we screened were successfully profiled, our study points to possible limitations for the RNA-tagging method that could stem from the binding location or binding mode of the protein of interest on each target (Hogan et al. 2008).

Further analysis focused on eight of these mRBPs, where we investigated the impact of deleting regions with RBDs on mRNA binding and on specificity. For mRBPs with clustered RBDs, such as Puf3, Puf4, Puf5, and Mrn1, deletion of these domains resulted in a complete loss of mRNA binding for their respective targets, indicating the indispensability of these RBDs and consistent with their strong evolutionary conversation. Conversely, for most mRBPs with dispersed RBDs, including Rie1, Nab6, and Scp160, deletion of individual RBDs led to a loss of specificity but not general binding ability as they lost some of their original targets while gaining new ones. This combined with their strong evolutionary conversation suggests that although possible, a change of binding specificity through individual RBD evolution is probably relatively rare. On the other hand, we observed weaker mRNA binding for Pub1 in each of its RBD deletions.

Moreover, we conducted complementary deletion analysis of non-RBD regions, enriched in extended IDRs, within these eight mRBPs. Interestingly, we found that determinants of binding and specificity were also present within these conserved extended IDRs. This is also true for IDRs located outside of the RBDs, refuting the possibility that they act only via positioning the RBDs. Complete abolishment of binding was observed upon deleting a highly disordered region flanking two structured RBD regions in Pub1 and the highly disordered, N-terminal non-RBD region in Mrn1. For Mrn1, the disordered region deleted is, however, located outside of the annotated RBDs suggesting a binding contribution. In general, deletion of non-RBD regions may influence binding specificity directly or indirectly via perturbing interactions with other proteins including RBPs.

In the case of Puf3, Puf4, and Puf5, deletion of highly disordered regions in their non-RBD often led to a reduction in their specificity and acquisition of new non-WT targets. Moreover, deletion of the most disordered region of ∼300AA within the non-RBD of Puf4 resulted in a complete loss of binding targets, although the RBD of Puf4 remained sufficient for binding and specificity in the context of the non-RBD of Puf3, suggesting a complex interplay between disordered binding determinants in Puf4's non-RBD and its RBD.

Overall, our results demonstrate that the binding of mRBPs to their mRNA targets relies on multiple determinants distributed throughout the protein sequence. This underscores the significance of the interplay between structured RBDs and non-RBD regions enriched in IDRs for achieving specificity and binding. As a similar interplay was observed in TFs (Brodsky et al. 2020; Kumar et al. 2023), where it contributes to the structure and evolution of the gene regulatory network (Gera et al. 2022), it is tempting to speculate that this also applies to RBPs and posttranscriptional gene regulation.

## MATERIALS AND METHODS

### Strains and growth conditions

All *S. cerevisiae* RNA tagging strains of RBPs used for all experiments (target identification) were grown in SD media, and RNA was extracted in log-grown conditions.

### Construction of Pup2-tagged strains

Strains for RNA tagging with endogenously expressed RBP-Pup2 fusion proteins were generated by swapping the Swat acceptor module of the respective C-Swat library strain (Meurer et al. 2018) for the *C. elegans* Pup2 ORF (kind gift from Marvin Wickens) using CRISPR-based gene editing (Anand et al. 2017) as described in Gera et al. (2022). All strains with genotypes generated in this study can be found in Supplemental Table S1.

### Constructions of RBP mutant strains

Domain deletion strains were created on the background of Pup-2 fused RBPs, using CRISPR-based gene editing with a guide RNA against the deleted protein region and an 80 nt repair ssDNA homologous to the adjacent 40 bp on each side. For Puf3 non-RBD with Puf4 RBD (Fig. 5B,C), gRNA was targeted against the replaced RBD, and the repair was generated by amplifying Puf4 RBD using primers with 40 bp homologous overhang to their new location. All strains with genotypes generated in this study can be found in Supplemental Table S1.

### RNA extraction and library construction

To profile RNA binding targets of RBP-Pup2 strains, we used the RNA-tagging method (Lapointe et al. 2015). Total RNA from exponential growing RBP-Pup2 strains, containing tagged and untagged mRNAs, was extracted using the Machery Nagel kit and stored at −80°C for later use in library preparation. mRNA libraries were constructed through five steps: (1) Double poly(A) selection for the enrichment of mRNAs (tagged and untagged). (2) Ligation of barcoded RNA adapter with UMI to the 3′end of all transcripts [after poly(A) and potential poly(U) stretches]. (3) Reverse transcription using an RNA-adapter-specific primer, cDNA of transcripts contains full 3′ end [poly(A) and eventual poly(U) stretches]. (4) Tn5 cleavage and second adapter insertion into cDNA/RNA hybrid for amplification. (5) Amplification of the library with Tn5 and RNA adapter-specific primers. Steps 1–3 and 4–5 are adapted from Wiener et al. (2021) and Chapal et al. (2019), respectively (see Supplemental Fig. S3).

The concentration and fragment size of generated libraries were measured with Qubit (Thermo Fisher) and TapeStation (Agilent). Libraries were paired-end sequenced (at least 25 nt from each side) on NovaSeq6000 with at least 200,000 reads per sample.

### Data processing

After Tn5-barcode demultiplexing with bcl2fastq, adapter removal was used to demultiplex based on the internal (RNA-adapter) barcode. After filtering adapter dimers using Cutadapt, bowtie2 was used to align read 1 (5′ of mRNA) to the yeast genome (64-1-1), and a custom script was used to extract and count the number of post-poly(A) Us on read 2 (reverse complement). U-count matrix for genes (rows) within the U stretches of different lengths (columns, 0–16 Us) were then generated using bedtools (coverage function), only counting sense transcripts around the transcription termination site of each gene (300 nucleotides [nt] upstream and 100 nt downstream). U-count matrixes are then imported to MATLAB for normalization and further analysis. U-count matrixes were normalized to sample size by scaling to a total number of one million counts. RPM normalized matrices of repeats with more than 200,000 reads or high median of binding reliability (see below) were combined for each sample (ordered U-count matrix of Puf3 is presented in Fig. 2A).

### RBP sequence of the *S. cerevisiae* proteome (Fig. 1A–D)

We first extracted the names of all RNA and mRBP-binding proteins from SGD and all TFs from CIS-BP (Weirauch et al. 2014). Then we collected all Pfam domain annotation (type and location) for each protein from SGD and only kept those with a well-defined RBD or DBD (see Supplemental Table S2), and further filtered out those RBPs that belonged to a bigger complex like the spliceosome or the ribosome, leaving 81 mRBPs, 88 genRBPs, and 156 TFs (see Fig. 1A). For each of these proteins, we then also calculated the disorder tendency along the full sequence using IUPred2 (Mészáros et al. 2018). For Figure 1A, the combined length of all DBDs or RBD in each protein is calculated based on the Pfam annotation, and the length of the IDR is calculated as those residues outside the DBD/RBD with a disorder tendency >0.5. For Figure 1B,C, Pfam RBDs (Mistry et al. 2021) from SGD (Cherry et al. 2012) were classified into 12 groups, and the rest combined into “others” (see Supplemental Tables S3 and S4). For Figure 1D, the AA composition of all IDR residues (outside of RBD/DBD and disorder tendency > 0.5), was determined and the pairwise Pearson correlation between the IDR-composition vector of all proteins was calculated. In the heatmap, proteins are ordered according to their similarity with the average TF composition. Of note, chosen mRBPs are a subgroup of mRBPs.

### mRBP sequence conservation across Saccharomycetaceae (Fig. 1E,F)

For each mRBP, the sequence of orthologs from 20 Saccharomycetaceae species, including post-WGD and non-WGD species, was extracted from YGOB (Byrne and Wolfe 2005) (hereafter called the ortholog group). Then, for each ortholog, we determined the disorder tendency across the full sequence using IUPred2 (Mészáros et al. 2018) and defined all Pfam domains (Mistry et al. 2021) using Hmmer (Potter et al. 2018), keeping only those known to bind RNA and ordering them based on global multiple sequence alignments of all proteins. Afterward, for each ortholog group, we defined those RBDs present in more than 30% of orthologs as the ancestral domain composition. We then checked for each of the orthologs in an ortholog group if it contained all ancestral domains in the right order. The fraction of these orthologs with conserved domain organization is shown in Figure 1E. We further calculated the mean domain score across all domains, and the average sequence similarity between a domain across different orthologs as shown in Supplemental Figure S2. To analyze IDR conservation, we first calculated the number and fraction of disordered residues in each non-RBD segment of every ortholog. We then defined IDR segments as those segments that have an average disorder tendency >0.5 across all orthologs and an average length of 15 or more residues. For each IDR segment, we then calculated the average and standard deviation of the number of disordered residues across the ortholog group, considering only those orthologs with the conserved domain organization. The number and average ratio (std/mean) of IDR segments in each ortholog group are shown in Figure 1F.

### U-counts correlation (e.g., binding reliability) (Figs. 2, 4, 5)

To estimate the internal consistency and reliability of RNA-tagging data in each sample, we calculated the pairwise Pearson correlation of U counts across all genes between U stretches of length 0–16 (i.e., columns in the normalized read count matrix). Sample tagging reliability corresponds to the correlation across all genes between U counts of length 4 and 13 in one sample. Intrasample median binding reliability was calculated based on the sample tagging reliability; the median was calculated for the best correlation of each U stretch with any other U stretch (Fig. 2C).

### Expression level, binding score, and modification level

Throughout the analysis, expression was calculated as the sum of all U counts (0–16) for each gene. $exp(gene)=\sum_{u=0}^{16}$ RPM(u,gene), with exp(gene) being the transcript abundance. Throughout the analysis, the gene-specific binding score was calculated based on the weighted sum of U counts of U stretches of length 4–13 for each transcript. bs(gene)= $\sum_{u=4}^{13}u*RPM(u,gene)$, with bs(gene) being the gene-specific binding score and RPM(u, gene) being the normalized read count for gene and a specific number of Us. Figure 3 compares both measures for each sample in the log2 scale. The ratio of binding score to expression is defined as the modification level.

### Target determination and GO-term analysis (Figs. 3–5)

To determine RBP targets, we first calculated the modification level (ratio of binding score to expression) in the log2 scale (see above). Transcripts with a ratio higher than the background ratio of dPup2 sample: −2.7 (log2 scale, 0.15 in linear scale) were defined as targets. Significantly enriched GO slim terms among targets were determined with the hypergeometric test function of MATLAB (hygecdf) and all expressed genes as a background.

### Correlation of deletion strains with WT

To compare the binding profile of RBP mutants with the WT, the modification level across all genes in the log2 scale was calculated, and each mutant was correlated with WT using this measure.

## DATA DEPOSITION

All raw sequencing data obtained during this study can be found on GEO (GSE235209). Python and Matlab scripts to process the data and generate the figures are available on GitHub (https://github.com/barkailab/zigdon2023).

## SUPPLEMENTAL MATERIAL

Supplemental material is available for this article.
